# A comparative study of environmental responsibility behavior in ecotourism from the perceptions of residents and tourists: A case of Qilian Mountains National Park in China

**DOI:** 10.1371/journal.pone.0281119

**Published:** 2023-02-24

**Authors:** Yaobin Wang, Ruitao Zhao, Zhenbing Yan, Meizhen Wang, Yinggang Pan, Ruoxue Wu

**Affiliations:** School of Tourism, Northwest Normal University, Lanzhou, China; University of Baltistan, PAKISTAN

## Abstract

The environmentally responsible behaviors of residents and tourists are great significance to the protection of natural resources and sustainable development of ecotourism. This paper takes China’s Qilian Mountains National Park as the case place. By constructing a theoretical model of perceived value on environmentally responsible behavior and studying the relationship between residents’ and tourists’ perceived value, satisfaction and environmentally responsible behavior from both subject and object perspectives, the study shows that. Educational level and occupational distribution have significant effects on residents’ and tourists’ perceptions of ecotourism environmentally responsible behaviors, but age only has a significant effect on residents’ perceptions of ecotourism environmentally responsible behaviors. Gender differences do not affect residents’ and tourists’ perceptions of ecotourism environmentally responsible behaviors. The theoretical model between residents’ perceptions of environmentally responsible behaviors, environmentally responsible behaviors, and satisfaction was basically confirmed. Perceived environmentally responsible behaviors of tourists does not affect satisfaction. Satisfaction has a positive effect on tourists’ environmentally responsible behaviors. Perceived environmental responsibility of tourists has a significant positive effect on tourists’ environmentally responsible behaviors. The overall level of residents’ perception of environmentally responsible behaviors in ecotourism is higher than tourists’ perception. Residents and tourists have a poor perception of ecological and environmental protection policies. This paper expects to strengthen residents’ and tourists’ perceptions of ecologically responsible behaviors. Establishing the sentiment of satisfaction and commitment to environmental protection motivates residents and tourists to implement environmentally responsible behaviors and contribute to the sustainable development of ecotourism.

## 1. Introduction

National parks are of vital ecological strategic significance to the protection of resources and environment and the sustainable development of tourism. While enjoying the natural scenery, tourists took responsibility for protecting nature and reduced the environmental impact of their activities [1]. At the same time, the funds from consumption in ecotourism areas were used to protect ecological areas with natural resources [2]. Ecotourism is growing at an annual rate of 20–25%, making it the fastest growing tourism product in China [3]. However, with the surge in the number of tourists, the uncivilized behavior of tourists has caused a certain degree of damage to the natural environment of the national parks, affecting the perceived behaviors of the residents [4]. As participants in tourism activities and subjects of ecological environment protection, residents’ and tourists’ environmental responsibility behaviors are an effective way to reduce the negative impact of recreation and enhance the sustainable development ability of ecotourism in national parks [5]. Achieving sustainable development in tourist destinations requires fostering and improving the implementation of environmentally responsible behaviors by residents and tourists [6]. Zhang [7] found that a satisfying tourism experience can enhance the environmentally friendly behaviors of tourists. Perceived value is considered an important influential factor in improving tourist satisfaction [8, 9]. Chiu et al. [10] found that tourist satisfaction not only significantly enhances pro-environmental behaviors and influences tourist perceptions. Zhuang et al. [11] found that residents, as social individuals closely related to tourist destinations, have positive or negative value perceptions of tourism development, which influence residents cognition, attitude and behaviors [12]. Guo et al. [13] confirmed the influence of the perceptions of the residents of the ancient town tourism sites on their behaviors. Zhu et al. [14] explored the impact of residents’ perceptions of tourism development on their life satisfaction based on social exchange theory. Yi et al. [15] argued that investigating residents’ pro-environmental behaviors and satisfaction is an important prerequisite basis for developing destination tourism and carrying out tourism planning.

Scholars have analyzed the perceived value, satisfaction and behavioral intentions of residents and tourists in different areas [16, 17], but most studies have been conducted from a single perception perspective of residents or tourists. There is a lack of studies comparing the relationship between residents’ and tourists’ environmentally responsible behaviors, perceptions and satisfaction from both host and guest perspectives, and there is a lack of theoretical support for perceptions of environmental responsibility and satisfaction. There is an urgent need for an effective theory to guide the research process to achieve sustainable ecotourism. The theory of planned behavior explains the mechanism of action by which perception influences behaviors and provides a basis for understanding the relationship between residents and tourists’ perceptions and behaviors. Emotional adaptation theory holds that an individual may not react satisfactorily or unsatisfactorily until they perceive something, which helps provide insight into the relationship between perception and satisfaction.

In conclusion, this paper takes the Qilian Mountains National Park in China as the research object and compares the relationship between residents’ and tourists’ environmentally responsible behaviors, perceptions, and satisfaction based on a dual perspective of host and guest and the theory of planned behavior and emotional adaptation. This paper expects to strengthen residents’ and tourists’ perceptions of eco-responsible behaviors, establish satisfaction and responsibility for environmental protection, encourage residents and tourists to implement environmentally responsible behaviors and contribute to the sustainable development of ecotourism.

## 2. Literature review

### 2.1 Environmentally responsible behaviors

Research on environmentally responsible behaviors dates to the 1970s, when Borden [18] defined it as actions taken by individuals or groups to improve the environment. Lehman [19] considered environmentally responsible behaviors as the behavior and efforts made by individuals to protect the environment, emphasizing environmentally conscious participation and concern. Kollmuss [20] defines it as an act by an individual or organization to protect the environment by working to have a minimal impact on the environment. With the development of research, environmentally responsible behaviors have been applied to tourism research. Xu [21] divides tourism environmental responsibility behaviors into two dimensions: self-discipline behaviors and conservation-promoting behavior. Lee [22] divides environmental responsibility behaviors into physical behaviors, pro-environmental behaviors, civic behaviors, environmentally friendly behaviors, persuasive behaviors, financial behaviors, and sustainable behaviors. Luo et al. [23] defined environmental responsibility behaviors as proactive actions performed by individuals based on their values and sense of responsibility that contribute to environmental protection and sustainable, and extended the concept of environmental responsibility behaviors to tourists. Zhang et al. [24] considered environmental responsibility behaviors as necessary actions taken by individuals for environmental protection that contribute to the health of the environment. Cai [25] argues that environmental responsibility behaviors are the result of the interaction between tourism activities and the environment and are essential for the ecological sustainability of tourism destinations and sustainable tourism development. Lee [26] argued that residents’ environmental responsibility behaviors refer to the various behavioral activities that residents exhibit in the face of a series of environmental changes and environmental problems with specificity and relevance.

This paper defines environmental responsibility behavior as the behavior of residents and tourists to maintain the natural ecological environment of tourist sites under certain tourism conditions, which is beneficial to the environmental health of the tourist place.

### 2.2 Satisfaction

Research on satisfaction in tourism science dates to the 1970s and refers to customer-centered research on consumer attitudes and prediction of behavioral activities based on customer satisfaction [27]. Pizam [28] considers the gap between tourists’ expectations before the tour and their feelings after the experience as tourist satisfaction. Later scholars have also mostly borrowed from Pizam and enriched and refined it, contributing to the development of the connotation of tourism satisfaction. Barker [29] pointed out that tourist satisfaction is a collection of tourists’ perceptions of the tourism environment, infrastructure, and services in a comprehensive evaluation. Li [30] argues that tourist satisfaction is a sense of pleasure that can be enhanced by a evaluation of the perception of the tourism environment. Bosque [31] believe that the personal cognitive and emotional states generated by tourism experience are called satisfaction. Swan [32] further enriched the meaning of satisfaction by dividing perceptions into tourism input perceptions and tourism output perceptions through a social exchange perspective of "inputs and outputs". When tourism input perceptions are smaller than tourism output perceptions, tourism participants become satisfied, and vice versa, dissatisfied. Francken et al. [33] assessed satisfaction based on a tourist experience perspective and concluded that the difference between tourists’ perceptions of ideal and actual tourism experiences determines the level of satisfaction. Zhang [34] explored the definition of satisfaction, which he considered to be a comprehensive psychological evaluation. Chen & Tsai [35] believes that satisfaction is the total pleasure of visiting a destination. Han [16] defines satisfaction as the degree to which perceptions of experience compare with tourism expectations.

This paper defines satisfaction as the degree of satisfaction of residents or tourists with the current state of the local ecotourism environment. Only by developing a tourism industry that meets the satisfaction of residents and tourists can local governments develop tourism in a more locally appropriate and sustainable manner.

### 2.3 Tourism environment perception

The study of perception in tourism studies dates to the 1980s, Morrison [36] considered tourist perceived value as a psychological evaluation of the tourism product determined by tourists after assessing the costs they pay and the benefits they obtain. Stevens [37] considered tourism perception as the result of tourists’ evaluation of the quality and level of service after experiencing tourism services. David [38] analyzed the perceptions of the residents of tourist places and found that the residents of cities with more rapid tourism development have negative attitudes toward the development of tourism. With the development of research, scholars begun to explore tourism environment perception. Bai [39] believes that tourism perception is the awareness and evaluation of the tourism environment and services formed by residents and tourists after comparing the tourism information they received at the tourist places with their own experiences. Cheng et al. [40] consider that tourism environment perception is the fundamental factor that restricts and regulates tourism behavior. Zhang et al. [41] argue that tourism environmental responsibility perception is the condition and basis for tourists and residents to implement environmentally responsible behaviors. Zhu and Lubell [42, 43] consider the perception of environmentally responsible behaviors in tourism as the perception that an individual’s behaviors in tourism activities are influenced by the behaviors of other individuals, thus regulating their behaviors in implementing environmental resource conservation.

## 3. Research hypothesis

### 3.1 Hypothesis of the relationship between perceived environmentally responsible behaviors and satisfaction

Emotional adaptation theory suggests that individuals may react to an event in a satisfactory or unsatisfactory manner only after perceiving it. In the research context of this paper, residents and tourists will develop certain perceptions of environmentally responsible behaviors concerning the natural environment and personnel activities that they are exposed to or perceive during ecotourism. The harmonious environmental protection atmosphere and beautiful environment in the scenic spot inspire the enthusiasm of residents and tourists and improve their satisfaction. Several studies have been conducted to identify the role of environmental perceptions in influencing satisfaction. Hao’s research [44] confirmed that perceptions of the tourist environment directly affect the lives of residents and the satisfaction of tourists with tourism. Huang et al. [45] found that by increasing residents’ and tourists’ perceptions of the tourism environment, they could enhance their satisfaction with tourism activities. Gong et al. [46] found that the higher the tourist’s perception of tourism, the higher their satisfaction. Similar findings were found by Zhang et al. [47] in their study of residents. Based on the above analysis and research basis, the following hypotheses are proposed in this paper.

H1: Residents’ perception of environmentally responsible behaviors has a positive effect on satisfaction
H2: Perceived environmentally responsible behaviors of tourists have a positive effect on satisfaction

### 3.2 Hypothesis of the relationship between satisfaction and environmentally responsible behaviors

Satisfaction is a positive affective attitude of residents or tourists arising from their participation in tourism activities. Numerous studies in the field of tourism confirm the relationship between satisfaction and positive behaviors of residents and tourists. When residents and tourists develop satisfaction emotions during their participation in tourism activities, they take the responsibility to protect the environment and implement behaviors that are beneficial to the environment. López-Mosquera & Sánchez [48] showed that the higher the tourist satisfaction with the destination, the higher the tendency to have environmentally responsible behaviors in most cases and the more pro-environmental positive behaviors. Davis et al. [49] found that tourists who are more satisfied with the destination are more willing to spend time and effort in the natural conservation of the destination. Chiu et al. [10] scholars confirmed that tourist satisfaction can significantly enhance their pro-environmental behavior. He et al. [50] confirmed from the perspective of community involvement that the higher the satisfaction of residents, the higher their willingness to engage in environmentally responsible behaviors. Wang et al. [51] found that the more satisfied residents are with the environment of a tourist destination, the more willing they are to engage in environmentally responsible behaviors through a study of the residents. Although the above research background on the relationship between satisfaction and environmentally responsible behaviors varies, most studies show that tourist satisfaction is an important predictor of environmentally responsible behaviors. A high level of satisfaction implies immediate or future positive environmental protection behaviors on the part of tourists. Therefore, the following hypothesis is proposed in this paper.

H3: Satisfaction has a positive effect on residents’ environmentally responsible behaviors
H4: Satisfaction has a positive effect on tourists’ environmentally responsible behaviors

### 3.3 Hypothesis of the relationship between perceived environmentally responsible behaviors and environmentally responsible behaviors

The theory of planned behavior assumes that an individual’s perceptions influence behavior norms. Individual behavior is not completely independent and in many cases is influenced by external factors. Environmental perceptions and policy perceptions can influence environmentally responsible behaviors. Residents and tourists facing a beautiful natural environment will develop a desire to protect it and pay attention to the impact of their behaviors on the environment. When the people in the scenic area perform environmentally responsible behaviors by example and protect the environment, individuals in the environment will also consciously obey the practice of protecting the environment. There is a correlation between residents’ and tourists’ perceptions of environmental responsibility and their implementation of environmentally responsible behaviors. Ming [52] found that awareness of environmental responsibility in tourism is the condition and basis for tourists and residents to carry out environmental responsibility. Wang et al. [53] found that public environmental facilities in scenic spots can promote environmentally responsible behaviors among tourists. Li et al [54] found a positive contribution of perceived environmental services in scenic areas to environmentally responsible behavior. Xia et al. [55] found that when residents live in places with beautiful environments, they have a sense of pleasure and pride in their hearts and take the initiative to take responsibility for protecting the environment. Huang [56] also found similar findings in a study of tourists. Based on the above analysis and research basis, the following hypothesis is proposed in this paper.

H5: The perception of residents’ environmentally responsible behavior has a positive effect on residents’ environmentally responsible behaviors
H6: Perceived environmentally responsible behavior of tourists has a positive influence on tourists’ environmentally responsible behaviors

**Fig 1:**
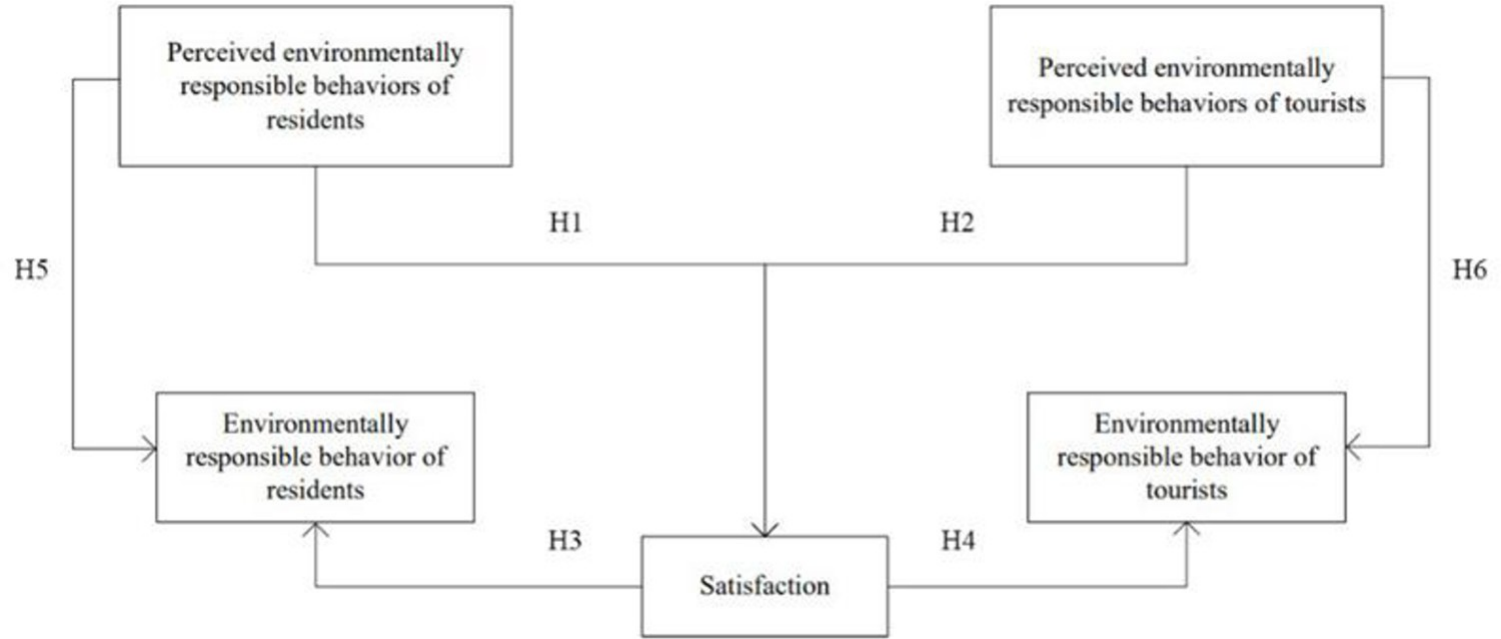
Research theoretical models.

To verify the above assumptions, a theoretical model is constructed, as shown in Fig 1.

## 4. Study design and data collection

### 4.1 Case site selection

**Fig 2:**
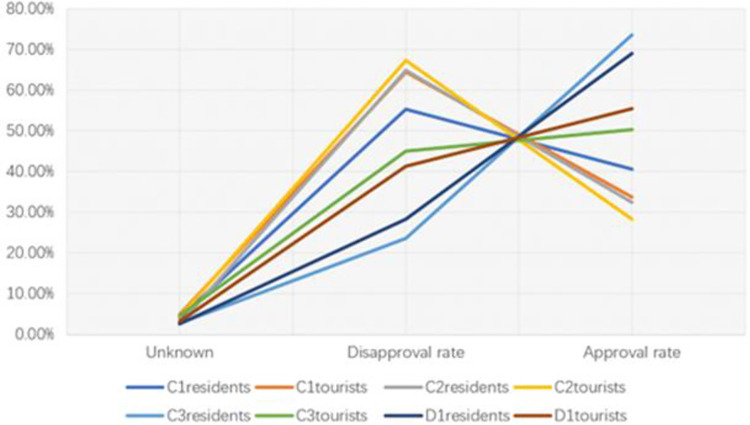
Comparison of C1, C2, C3, D1 options.

China’s Qilian Mountains National Park (Fig 2) is located at the junction of Qinghai and Gansu provinces with an altitude of 1684–5604 meters. It is surrounded by the Qinghai-Tibet Plateau in the south and the Hexi Corridor in the north, with diverse natural ecosystems and abundant wildlife resources. It is an extremely important ecological functional area for glacier and water conservation, wildlife migration corridors, biological special resource banks and genetic gene banks in China. It is also a key ecological function area and a barrier to ecological security in China. The park generally runs from northwest to southeast, with a narrow and long area. Its total area is 5.02×104km^2^, with eight nature reserves, a core protection area of 27,500 square kilometers and a general control area of 22,700 square kilometers. The protected area is home to 53,243 residents in 14 counties.

### 4.2 Questionnaire design

Referring to scholars on the scale of ecotourism environmental responsibility behaviors perceived by residents and tourists [57–59], combined with the actual situation of the case site, the questionnaire was designed. The questionnaire was based on recognized scales of knowledge and concern for the ecological environment. Questions on environmental responsibility were added. The resident and visitor questionnaires included demographic characteristics (sex, age, education, occupation), overall perception of ecotourism environment, perception of ecological environmental protection policy and perception of ecotourism environment in Qilian Mountains National Park. Since residents and tourists have different perceptions of the environmental impact of ecotourism, the two questionnaires asked different questions, and both were measured using the Likert scale. Table 1 gives the definition and measurement options of the research dimensions.

**Table 1:** Study construct definitions and measurement items.

Construct	Construct definitions	Measurement items
Overall perception of ecotourism environment	Positive or negative evaluation of the overall ecotourism environment by residents and tourists	A_1_ Ecotourism promotes the protection of local natural resources and environment
		A_2_ Ecotourism raises environmental awareness among residents and governments
		A_3_ Ecotourism generates more domestic waste
Environmental perception of ecotourism in Qilian Mountains National Park	Positive or negative evaluation of the ecological environment and ecotourism development in Qilian Mountains National Park by residents and tourists	B_1_ Qilian Mountain National Park has a strong ecological protection
		B_2_ The current state of development of ecotourism in Qilian Mountains National Park is satisfactory
Ecological environmental protection policy perception	Satisfaction level of residents and tourists with the government and scenic spots’ ecological and environmental protection policies	C_1_ The government has invested more supervision in ecological environment governance
		C_2_ The existing laws and regulations on ecotourism and environmental protection are robust
Perceived environmentally responsible behaviors of residents	Positive or negative comments from tourists about residents or residents about residents’ environmentally responsible behaviors	D_1_ Some residents who participate in tourism only pay attention to economic income and do not care about ecological protection
		D_2_ Some residents do not follow the environmental protection rules and orders of Qilian Mountain National Park
		D_3_ Residents will participate in environmental protection activities in ecological scenic spots
		D_4_ The uncivilized behavior of residents will seriously affect the ecological environment
		TD_1_ Residents will take the initiative to clean up the garbage in ecological scenic area
		TD_2_ Residents will discourage or stop tourists from non-environmentally friendly behavior
Perceived environmentally responsible behaviors of tourists	Positive or negative comments from residents about tourists or tourists about tourists’ environmentally responsible behaviors	E_1_ Tourists protect the ecological environment during the tour
		E_2_ Tourists keep the environmental rules of the ecological scenic spot during the tour
		E_3_ Tourists will provide advice to relevant departments in a timely manner when they discover environmental problems during the tour
		E_4_ Tourists actively promote the concept of environmental protection during the tour
		E_5_ Tourists will follow relevant environmental protection advice during the tour
		QE_1_ Tourists will participate in environmental protection activities in ecological scenic spots
		QE_2_ The uncivilized behavior of tourists will seriously affect the ecological environment

Note: The TD item is a unique item in the tourist questionnaire, and the QE item is a unique item in the resident questionnaire.

### 4.3 Data collection

To verify the scientific validity of the questionnaire, a pilot test of residents and visitors of China’s Qilian Mountains National Park was conducted. In the pilot test, 50 questionnaires were distributed to residents and 60 to tourists, 47 and 55 were returned respectively. After screening, 43 valid questionnaires for residents and 52 valid questionnaires for tourists were obtained, and the effective recovery rates were 86.00% and 86.67%. The reliability of the questionnaire was tested by CITC and Cronbach’s alpha. The test results for residents and tourists are shown in Table 2.

**Table 2:** Questionnaire reliability test results for residents and tourists.

Construct	Measurement items	CITC		Deleted Cronbach’s α	
		Residents	Tourists	Residents	Tourists
Overall perception of ecotourism environment	A_1_ Ecotourism promotes the protection of local natural resources and environment	0.505	0.457	0.815	0.829
	A_2_ Ecotourism raises environmental awareness among residents and governments	0.475	0.575	0.808	0.813
	A_3_ Ecotourism generates more domestic waste	0.275	0.168	0.716	0.849
Environmental perception of ecotourism in Qilian Mountains National Park	B_1_ Qilian Mountain National Park has a strong ecological protection	0.641	0.409	0.817	0.820
	B_2_ The current state of development of ecotourism in Qilian Mountains National Park is satisfactory	0.471	0.321	0.808	0.814
Ecological environmental protection policy perception	C_1_ The government has invested more supervision in ecological environment governance	0.367	0.442	0.812	0.809
	C_2_ The existing laws and regulations on ecotourism and environmental protection are robust	0.476	0.411	0.807	0.810
Perceived environmentally responsible behaviors of residents	D_1_ Some residents who participate in tourism only pay attention to economic income and do not care about ecological protection	0.364	0.436	0.812	0.813
	D_2_ Some residents do not follow the environmental protection rules and orders of Qilian Mountain National Park	0.467	0.455	0.822	0.807
	D_3_ Residents will participate in environmental protection activities in ecological scenic spots	0.382	0.435	0.811	0.808
	D_4_ The uncivilized behavior of residents will seriously affect the ecological environment	0.274	0.221	0.736	0.745
	TD_1_ Residents will take the initiative to clean up the garbage in the ecological scenic area		0.462		0.807
	TD_2_ Residents will discourage or stop tourists from non-environmentally friendly behavior		0.439		0.813
Perceived environmentally responsible behaviors of tourists	E_1_ Tourists protect the ecological environment during the tour	0.603	0.565	0.861	0.864
	E_2_ Tourists keep the environmental rules of the ecological scenic spot during the tour	0.558	0.864	0.674	0.855
	E_3_ Tourists will provide advice to relevant departments in a timely manner when they discover environmental problems during the tour	0.628	0.859	0.634	0.858
	E_4_ Tourists actively promote the concept of environmental protection during the tour	0.246	0.139	0.797	0.713
	E_5_ Tourists will follow relevant environmental protection advice during the tour	0.197	0.294	0.815	0.832
	QE_1_ Tourists will participate in environmental protection activities in ecological scenic spots	0.466		0.855	
	QE_2_ The uncivilized behavior of tourists will seriously affect the ecological environment	0.598		0.849	

Note: The TD item is a unique item in the tourist questionnaire, and the QE item is a unique item in the resident questionnaire.

The 20 items of the pilot test were tested for reliability, and the initial reliability of the questionnaire for residents and tourists was 0.742 and 0.661, the CITC values of A_3_, D_4_, E_4_, and E_5_ were all less than 0.3. After the above items were deleted, the reliability coefficients were 0.836 and 0.854. The reliability values after removal were higher than the initial values, and the items were deleted from the process. To have a more comprehensive perception of ecological environmental protection policy, item C_3_ was added. The test results are shown in Table 3.

**Table 3:** C_3_ reliability test results.

Construct	Measurement items	CITC		Deleted Cronbach’s α	
		Residents	Tourists	Residents	Tourists
Ecological environmental protection policy perception	C_3_ The existing ecological and environmental management technology is highly innovative	0.392	0.433	0.811	0.809

According to the analysis of the questionnaire results, C_3_ has a good reliability. The 17 items of the questionnaire do not meet the deletion conditions, have a good reliability, and meet the requirements for collection and research.

## 5. Case study analysis

### 5.1 Data sources and methods

Questionnaires were administered to residents and tourists of Qilian Mountains National Park using a random sampling method. The questionnaires were entrusted to regional-related personnel for distribution in January 2022, and the collection was completed in February 2022. A total of 876 valid questionnaires were collected, including 335 questionnaires for residents and 541 questionnaires for tourists. Using Spss23.0 and Amos23.0, linear regression analysis and path analysis tests were used to analyze the obtained questionnaire data to compare and study the relationship between residents’ and tourists’ perceptions of environmentally responsible behaviors, environmentally responsible behavior and satisfaction. Underage participants were asked to complete the questionnaire with the consent of their parents and themselves. The questionnaires in this paper were filled out voluntarily by the respondents. All respondents were informed and actively participated in the questionnaire completion process. Questionnaires completed by respondents can be used as supporting information.

### 5.2 Demographic profiles

Table 4 shows that in this questionnaire survey, the ratio of gender and age of residents and tourists were roughly the same, the distribution of education level and occupation reached the expected standard, and the demographic profile has a good degree of contrast. These all met the requirements of the survey.

**Table 4:** Demographic characteristics of residents and tourists.

Project	Project composition	Number of samples		Proportion %	
		Residents	Tourists	Residents	Tourists
Gender	Male	146	267	43.70%	49.35%
	Female	189	274	56.30%	50.65%
Age	Under 18	27	36	8.15%	6.65%
	18–29	117	237	34.81%	43.81%
	30–44	104	214	31.11%	39.56%
	45–59	67	39	19.98%	7.21%
	Over 60	20	15	5.95%	2.77%
Educational level	Bachelor’s degree or above	97	283	28.89%	52.31%
	Specialist/Higher Vocational	119	118	35.52%	21.81%
	Secondary school/High school	84	97	25.07%	17.93%
	Junior high school	21	37	6.27%	6.84%
	Elementary school and below	14	6	4.25%	1.11%
Occupational distribution	Government staff	5	69	1.48%	12.75%
	Teacher	12	23	3.70%	4.25%
	Corporate employees	77	166	22.96%	30.68%
	Student	60	238	17.78%	43.99%
	Farmers and pastoralists	122	21	36.30%	3.88%
	Retirees	17	11	5.19%	2.03%
	Others	42	13	12.59%	2.42%

### 5.3 Reliability test

To verify the internal consistency of each variable item, reliability tests were carried out for the construct in the questionnaires of residents and tourists. The results are shown in Tables 5 and 6.

**Table 5:** Reliability of residents’ questionnaire.

Construct	Cronbach’s α profile reliability	Cronbach’s α overall reliability
Overall perception of ecotourism environment	.866	.880
Environmental perception of ecotourism in Qilian Mountains National Park	.812	
Ecological environmental protection policy perception	.861	
Perceived environmentally responsible behaviors of residents	.756	
Perceived environmentally responsible behaviors of tourists	.859	

**Table 6:** Reliability of tourists’ questionnaire.

Construct	Cronbach’s α profile reliability	Cronbach’s α overall reliability
Overall perception of ecotourism environment	.826	.800
Environmental perception of ecotourism in Qilian Mountains National Park	.717	
Ecological environmental protection policy perception	.873	
Perceived environmentally responsible behaviors of residents	.768	
Perceived environmentally responsible behaviors of tourists	.801	

The reliability of the tourist questionnaire is 0.880, meaning that that the indicators of this questionnaire are consistent. The reliability of Cronbach’s α for each element is between 0.756–0.866, all greater than 0.7, indicating that the reliability of the tourist questionnaire is good and meets the requirements of internal consistency.

The reliability of tourist questionnaire is 0.800; the indicators of this questionnaire are therefore consistent. The reliability of Cronbach’s α for each element is between 0.717–0.873, all greater than 0.7, indicating good reliability of the tourist questionnaire and that it meets all criteria of internal consistency.

### 5.4 Sample dependent variable analysis

To verify whether sociodemographic characteristics can significantly change the measurement results, and whether there is an interaction among sociodemographic characteristics, this research conducted an independent samples t-test for the statistical characteristic option of gender and a one-way ANOVA for age, education, and occupation, while testing for differences in the correlations between the independent and dependent variables.

#### 5.4.1 The results of the questionnaire on residents’ perception of environmental responsibility

The results of the descriptive statistical analysis of resident questionnaire are shown in Table 7.

**Table 7:** Statistical analysis chart of resident scale.

	Sample size	Minimum	Maximum	Mean	Standard deviation	Low rate	High rate
Overall perception of ecotourism environment	335	1.00	5.00	4.25	0.87	12.1%	67.4%
Environmental perception of ecotourism in Qilian Mountains National Park	335	1.00	5.00	3.31	1.73	18.5%	59.2%
Ecological environmental protection policy perception	335	1.00	5.00	3.39	1.10	44.6%	35.3%
Perceived environmentally responsible behaviors of residents	335	1.00	5.00	3.72	0.87	30.4%	49.6%
Perceived environmentally responsible behaviors of tourists	335	1.00	5.00	4.04	0.76	11.9%	76.7%

Table 7 shows that residents’ perceptions of eco-environmental protection policies and their perceptions of residents’ environmentally responsible behaviors are more divergent in opinion. The residents’ overall perception of the ecotourism environment and the perceived level of environmentally responsible behaviors of tourists is weak, and there is still much room to improve the development of local ecotourism and meet the residents’ demands for ecotourism development. Therefore, it is very important to expand the positive impact of ecotourism by providing targeted countermeasures and improving the perception of residents. The government should take relevant measures to strengthen the guidance of ecological environment management and ensure the balance between environmental protection and tourism development, so that residents can enjoy the benefits brought by ecotourism, while encouraging them to actively participate in the act of ecological environmental protection.

Table 8 shows that by comparing the demographic characteristics of the residents while satisfying independence and chi-square, it is found that the demographic characteristics of the residents have a more significant effect on their perception of environmental responsibility. Gender differences do not differ significantly across constructs, which means resident gender does not affect the dimensional elements. Age, education level and occupation had a significant effect on residents perceived environmental responsibility impact of ecotourism. Therefore, it is necessary to survey residents of different ages, education levels and occupations.

**Table 8:** The test results of significant differences in resident questionnaire.

Testing items	Gender (two-way significance)	Age significance	Educational level significance	Occupation significance
Overall perception of ecotourism environment	0.413	0.584	0.039*	0.245
Environmental perception of ecotourism in Qilian Mountains National Park	0.205	0.823	0.012*	0.036*
Ecological environmental protection policy perception	0.944	0.009*	0.895	0.489
Perceived environmentally responsible behaviors of residents	0.647	0.330	0.246	0.016*
Perceived environmentally responsible behaviors of tourists	0.169	0.029*	0.043*	0.372

Note: significant P<0.05 is marked with *

As shown in Table 9, it is found through correlation analysis that, there is a significant positive correlation between the overall perception of ecotourism environment, environmental perception of ecotourism in Qilian Mountains National Park, ecological environmental protection policy perception, perceived environmentally responsible behaviors of residents and the perceived environmentally responsible behaviors of tourists. The ecotourism environment perception in Qilian Mountains National Park contains the situation of satisfaction with the ecological environment and ecotourism development. To explore significant influence of the positive correlation of other dimensions on satisfaction, the construct of ecotourism perception in Qilian Mountains National Park was converted into satisfaction. Referring to He [50], TD_1_ and TD_2_ were studied as residents’ environmentally responsible behaviors, and the resident model fitting indicators are shown in Table 10.

**Table 9:** Result of correlation analysis in residents’ questionnaire.

	Overall perception of ecotourism environment	Environmental perception of ecotourism in Qilian Mountains National Park	Ecological environmental protection policy perception	Perceived environmentally responsible behaviors of residents	Perceived environmentally responsible behaviors of tourists
Overall perception of ecotourism environment	1				
Environmental perception of ecotourism in Qilian Mountains National Park	.688**	1			
Ecological environmental protection policy perception	.220*	.221**	1		
Perceived environmentally responsible behaviors of residents	.240**	.351**	.582**	1	
Perceived environmentally responsible behaviors of tourists	.536**	.667**	.267**	.390**	1

Note

**correlation is significant at 0.01 (two-sided)

*correlation is significant at 0.05 (two-sided).

**Table 10:** Resident model fitting indicators.

Fitting index	*χ* ^2^	*df*	*χ*^2^/*df*	*GFI*	*AGFI*	*RMSEA*	*NFI*	*CFI*	*TLI*
Reference value			1<*χ*^2^/*df*<3	>0.900	>0.900	<0.800	>0.900	>0.900	>0.900
Actual value	239.742	196	1.739	0.936	0.915	0.332	0.937	0.985	0.924

The Amos 23.0 software was applied to perform the fitting. The specific fitting indexes are shown in Table 10. χ^2^ = 239.742, *df* = 196, χ^2^/*df* = 1.739, *GFI* = 0.936, *AGFI* = 0.915, *RMSEA* = 0.332, *NFI* = 0.937, *CFI* = 0.985, *TLI* = 0.924. All the fit indicators meet the judgment criteria, and the overall fit is good. The fit of the resident model to the data is at a better level, and the SEM hypothetical path can be checked and analyzed.

From Table 11, residents’ perception of environmentally responsible behaviors has a significant positive effect on satisfaction (p<0.01), and the path coefficient is 0.496, indicating that residents’ perception of environmentally responsible behaviors can improve the satisfaction level to some extent, and H1 passes the test. This may be because the residents feel the good ecological environment in the national park and have a higher level of perception to generate satisfaction. Satisfaction has a positive effect (p<0.05) on residents’ environmentally responsible behaviors with a path coefficient of 0.638, and hypothesis H3 holds, which is consistent with the findings of Tu [60] et al. The perception of residents’ environmentally responsible behaviors had a significant positive effect on residents’ environmentally responsible behaviors (P<0.01), with a path coefficient of 0.494, and H5 passed the test, which is consistent with the findings of Wang [61]. It is necessary to strengthen the publicity and education of ecotourism environmental protection, improve the level of residents’ ecotourism environmental perception and satisfaction, and improve environmental responsibility behavior. So that residents can participate more actively in ecological environmental protection behaviors.

**Table 11:** Resident model path analysis.

Path relationship	Standardized path coefficient	S.E.	C.R.	P
Perceived environmental responsible behaviors of residents → satisfaction	0.496	0.201	1.739	0.003
Satisfaction → Residents’ environmentally responsible behaviors	0.638	0.682	2.207	0.035
Perception of residents’ environmental responsibility → residents’ environmentally responsible behaviors	0.494	0.158	0.453	0.004

#### 5.4.2 The results of the questionnaire on tourists’ perception of environmental responsibility

The results of the descriptive statistical analysis of the tourist questionnaire are shown in Table 12.

**Table 12:** Statistical analysis chart of tourist scale.

	Sample size	Minimum	Maximum	Mean	Standard deviation	Low rate	High rate
Overall perception of ecotourism environment	541	1.00	5.00	3.54	1.03	13.3%	66.3%
Environmental perception of ecotourism in Qilian Mountains National Park	541	1.00	5.00	3.46	1.02	29.8%	41.6%
Ecological environmental protection policy perception	541	1.00	5.00	3.39	1.00	36.3%	48.2%
Perceived environmentally responsible behaviors of residents	541	1.00	5.00	3.12	0.74	22.7%	71.8%
Perceived environmentally responsible behaviors of tourists	541	1.00	5.00	3.46	0.99	15.4%	74.1%

Table 12 shows that tourists diverge in their perceptions of the ecotourism environment in Qilian Mountains National Park and the environmentally responsible behaviors of residents. The perceptions of tourists about the overall ecotourism environment, the perceptions of ecotourism environment in Qilian Mountains National Park, the perceptions of ecological environmental protection policies, the perceptions of residents’ environmentally responsible behaviors, and the perceptions of tourists’ environmentally responsible behaviors are not satisfactory, and the perceptions tend to be at a lower level. Therefore, it is very important to improve this perception. To strengthen the ecological protection of China’s Qilian Mountain National Park, the scenic spot should include environmental education in the planning of the scenic spot, to arouse the public’s ecological awareness and draw the attention of residents and tourists to the environment, thus contributing to the protection of the ecotourism area’s environment.

As shown in Table 13, while satisfying the independence and homogeneity of variance, by comparing the differences between different types of tourists, it is found that the demographic characteristics of tourists have a significant impact on tourists’ perception of environmental responsibility. Gender differences do not differ significantly across constructs and tourists’ gender differences do not affect the dimensional elements in this paper. Educational level and occupation have certain significant effects on tourists’ perception of environmental responsibility. Therefore, it is necessary to study tourists of different levels of education and occupations.

**Table 13:** The test results of significant differences in tourists’ questionnaire.

Testing items	Gender (two-way significance)	Age significance	Educational level significance	Occupation significance
Overall perception of ecotourism environment	0.563	0.812	0.978	0.006
Environmental perception of ecotourism in Qilian Mountains National Park	0.861	0.742	0.036*	0.016*
Ecological environmental protection policy perception	0.195	0.421	0.045*	0.374
Perceived environmentally responsible behaviors of residents	0.289	0.703	0.032*	0.684
Perceived environmentally responsible behaviors of tourists	0.936	0.898	0.314	0.372

Note: significant P<0.05 is marked with *

As shown in Table 14, it is found through correlation analysis that, there is a significant positive correlation between the overall perception of ecotourism environment, environmental perception of ecotourism in Qilian Mountains National Park, ecological environmental protection policy perception, perceived environmentally responsible behaviors of residents and the perceived environmentally responsible behaviors of tourists. Consistent with the conclusions drawn from the resident questionnaire. The same steps as the residents’ questionnaire scale analysis were used to convert the perception of ecotourism environment in Qilian Mountains National Park into satisfaction. Referring to the study of Lu [17] and Gong [46], QD_1_ and QD_2_ were studied as tourists’ environmentally responsible behaviors, and the tourist model fitting indicators are shown in Table 15.

**Table 14:** Result of correlation analysis in tourists’ questionnaire.

	Overall perception of ecotourism environment	Environmental perception of ecotourism in Qilian Mountains National Park	Ecological environmental protection policy perception	Perceived environmentally responsible behaviors of residents	Perceived environmentally responsible behaviors of tourists
Overall perception of ecotourism environment	1				
Environmental perception of ecotourism in Qilian Mountains National Park	.803**	1			
Ecological environmental protection policy perception	.211*	.362**	1		
Perceived environmentally responsible behaviors of residents	.645**	.735**	.396**	1	
Perceived environmentally responsible behaviors of tourists	.792**	.525**	.334**	.784**	1

Note

**correlation is significant at 0.01 (two-sided)

*correlation is significant at 0.05 (two-sided).

**Table 15:** Tourist model fitting indicators.

Fitting index	*χ* ^2^	df	*χ*^2^/*df*	GFI	AGFI	RMSEA	NFI	CFI	TLI
Reference value			1<*χ*^2^/*df*<3	>0.900	>0.900	<0.800	>0.900	>0.900	>0.900
Actual value	217.376	182	1.325	0.912	0.938	0.035	0.929	0.944	0.932

The Amos 23.0 software was applied to perform the fitting. The specific fitting indexes are shown in Table 15. χ^2^ = 217.376, *df* = 182, χ^2^/*df* = 1.325, *GFI* = 0.912, *AGFI* = 0.938, *RMSEA* = 0.035, *NFI* = 0.929, *CFI* = 0.944, *TLI* = 0.932. All the fitting indicators met the judgment criteria, and the overall fit was good enough for the SEM hypothetical path check and analysis.

As shown in Table 16, the effect of tourists’ perception of environmentally responsible behaviors on satisfaction failed the significance test (p>0.05), and hypothesis H2 was rejected, which is inconsistent with the findings of Dou [62]. It may be because the ecological resources in Qilian Mountains National Park deviate from tourists’ expectations, and there is a need to further explore the cultural connotation of the national park, to develop and utilize the core resources moderately based on ecological protection, and to improve the satisfaction level of tourists. Satisfaction has a positive effect on tourists’ environmentally responsible behaviors (p<0.05), with a path coefficient of 0.496, and hypothesis H4 passed the test. The reason for this may be that tourists’ satisfaction experience with the tourist place promotes the formation of their sense of identity, which in turn leads to the desire to protect the environment. Tourist perception of environmental responsibility had a significant positive effect on tourist environmentally responsible behaviors (p<0.01), with a path coefficient of 0.402 and H6 passing the test, consistent with the findings of Zhao [63]. By increasing tourists’ sense of responsibility for the environment in national parks, local government can stimulate their motivation to participate in environmental protection.

**Table 16:** Tourist model path analysis.

Path relationship	Standardized path coefficient	S.E.	C.R.	*P*
Perceived environmentally responsible behaviors of tourists → Satisfaction	0.023	0.132	2.903	0.774
Satisfaction → Tourists environmentally responsible behaviors	0.496	0.356	1.453	0.032
Tourist environmental responsibility perception → Tourist environmental responsibility behavior	0.402	0.113	1.739	0.007

#### 5.4.3 Comparative analysis of ecotourism responsibility in China’s Qilian Mountains National Park from the perspectives of hosts and guests

According to the Likert scale, 1<mean<2.5 is low, 2.5≤mean≤3.5 is medium, 3.5<mean≤5 is high. Table 17 shows that the overall perception of residents is more positive and obvious than tourists. The average level of tourists’ overall perception of the ecotourism environment exceeds 3.5, indicating that tourists are more optimistic about the development of ecotourism. In the five items, except for ecological environmental protection policy perception, the rest perception levels are above medium. The differences in perceptions of ecological environmental protection policies (p = 0.042, -0.08) and perceived environmentally responsible behaviors of residents (p = 0.031, +0.60) were more obvious between residents and tourists. In ecological environmental protection policy perception, residents have a dissatisfied attitude, tourists have a neutral attitude, and both residents (low level rate: 44.6%, high level rate: 35.3%) and tourists (low level rate: 36.3%, high level rate: 48.2%) are divided in their opinions. The reasons for this might be the weak implementation of local government regulations, unclear division of authority and responsibility between departments, neglect of goal-oriented ecological environmental protection, and lack of an effective environmental monitoring system, which makes the perception level of residents and tourists low. In the perception of residents’ environmentally responsible behaviors, both tourists and residents are neutral, residents (low level rate: 30.4%, high level rate: 39.6%), with more divergent opinions, and tourists (low level rate: 22.7%, high level rate: 71.8%), with less divergent differences. The reason for this might be that residents are more aware of specific local developments and better able to understand the specific problems of local development than tourists. Some residents do not have a solid concept of ecological civilization, and are obsessed with the pursuit of economic gains, ignoring the ecological and environmental problems brought about by economic development, bringing a series of negative impacts to the development of ecotourism in Qilian Mountains National Park.

**Table 17:** Contrastive dimensional analysis of perceived ecotourism environmental responsibility by residents and tourists.

Perceptual item	Mean		Increase or decrease	Standard deviation		Low rate		High rate		t	df	P(Bilateral)
	R	T		R	T	R	T	R	T			
Overall perception of ecotourism environment	3.25	3.54	-0.29	0.87	1.03	12.1%	13.3%	67.4%	66.3%	-2.79	195.54	0.376
Environmental perception of ecotourism in Qilian Mountains National Park	3.41	3.36	+0.05	1.73	1.02	18.5%	29.8%	59.2%	41.6%	-2.27	248.42	0.102
Ecological environmental protection policy perception	2.89	2.97	-0.08	1.10	1.00	44.6%	36.3%	35.3%	48.2%	-1.91	224.83	0.042*
Perceived environmentally responsible behaviors of residents	3.72	3.12	+0.60	0.87	0.74	30.4%	22.7%	39.6%	71.8%	-3.16	216.55	0.031*
Perceived environmentally responsible behaviors of tourists	4.04	3.46	+0.58	0.76	0.99	11.9%	15.4%	76.7%	74.1%	-5.51	262.19	0.512

Note: p<0.05 is marked with *, R (residents), and T (tourists).

Table 18 shows that the average value of perceptions of residents and tourists generally exceeds 3.0, which is in the medium and above medium level, and the perceived level is well. The perception level of residents about C_1_, C_2_, and C_3_ is poor, and the perceived level of tourists about C_1_, C_2_, and C_3_ is weaker than other tourist items, so it is necessary to conduct a detailed analysis (Fig 4.1). In D_2_, the average values of perceptions of residents and visitors are 1.41 and 1.12, indicating that residents follow the rules of the environmental protection order in the national park. In QE_1_ and QE_2_, the perceived average value exceeds 3.5, indicating that residents are agreeable to the environmentally responsible behaviors of tourists. In TD_1_, the perceived average value of tourists is 2.97, with widely divergent opinions (approval rate 59.6%, disapproval rate 36.3%), and local management should take measures to enable residents to take the initiative to clean up the garbage in scenic spots. In general, the perception level of residents is higher than that of visitors, which is consistent with the results of the survey analysis derived above.

**Table 18:** A comparative analysis of the ecotourism environmental responsibility perception perceived by residents and tourists.

Measurement options	Mean		Standard deviation		Disapproval rate (%)		Approval rate (%)	
	R	T	R	T	R	T	R	T
A_1_ Ecotourism promotes the protection of local natural resources and environment	3.51	3.42	1.054	.964	17.1	13.2	81.5	75.3
A_2_ Ecotourism raises environmental awareness among residents and governments	3.77	3.64	1.052	.887	13.4	11.8	83.7	77.6
B_1_ Qilian Mountain National Park has a strong ecological protection	3.18	3.96	1.102	.969	21.2	24.9	66.4	72.3
B_2_ The current state of development of ecotourism in Qilian Mountains National Park is satisfactory	3.43	3.25	1.167	1.138	27.2	26.4	59.2	54.4
C_1_ The government has invested more supervision in ecological environment governance	2.34	2.78	1.013	.947	55.3	61.4	40.6	33.7
C_2_ The existing laws and regulations on ecotourism and environmental protection are robust	2.41	2.98	.998	1.063	64.9	67.3	32.5	28.4
C_3_ The existing ecological and environmental management technology is highly innovative	3.55	3.03	1.362	.815	23.6	45.1	73.6	50.3
D_1_ Some residents who participate in tourism only pay attention to economic income and do not care about ecological protection	3.69	3.07	1.214	1.135	28.3	41.4	69.1	55.4
D_2_ Some residents do not follow the environmental protection rules and orders of Qilian Mountain National Park	1.41	1.12	.987	.911	69.2	57.8	19.3	24.7
D_3_ Residents will participate in environmental protection activities in ecological scenic spots	3.96	3.84	.724	.936	21.4	19.6	71.2	70.5
TD_1_ Residents will take the initiative to clean up the garbage in the ecological scenic area		2.97		.928		36.3		59.6
TD_2_ Residents will discourage or stop tourists from non-environmentally friendly behavior		4.13		.727		16.1		79.8
E_1_ Tourists protect the ecological environment during the tour	4.06	3.81	.809	.931	19.3	25.6	74.5	68.2
E_2_ Tourists keep the environmental rules of the ecological scenic spot during the tour	3.96	3.93	1.059	.960	24.4	27.9	71.3	69.1
E_3_ Tourists will provide advice to relevant departments in a timely manner when they discover environmental problems during the tour	4.12	4.25	.716	.431	13.2	17.6	78.2	80.6
QE_1_ Tourists will participate in environmental protection activities in ecological scenic spots	4.02		.786		17.9		76.9	
QE_2_ The uncivilized behavior of tourists will seriously affect the ecological environment	4.22		.322		10.2		86.3	

Note: The TD option is a unique option in the tourist questionnaire, and the QE option is a unique option in the resident questionnaire; R means residents, T means tourists.

According to Fig 2, residents and tourists have the same opinions on C_1_ and C_2_, the government’s investment and supervision in ecological environment governance are insufficient, and the public believes that the existing ecotourism environmental protection regulations are flawed. In C_3_, residents and tourists have very different opinions. The residents know more than tourists about ecological environment management technology. In D_1_, the approval rates of both residents and tourists are over 55%. Residents who participate in tourism activities pay more attention to tourism income than to environmental protection. The irresponsible environmental behaviors of residents may cause a series of ecological and environmental problems.

## 6. Conclusion and discussion

In the aspect of perception of ecotourism, residents have a limited perception. Gender differences among residents do not affect their questionnaire. Age, education and occupation distribution have a significant impact on residents’ perception of environmentally responsible behavior, which may explain why residents of different ages, education levels and occupations have different opinions on tourists’ environmentally responsible behaviors. In research, it is necessary to survey a diverse range of residents to ensure accuracy. In the correlation verification analysis, a significant positive correlation was found among the aspects in this study. Improving the correlation among aspects is key to improving the perception of tourists’ environmentally responsible behavior. The positive effect of residents’ perception of environmentally responsible behaviors on satisfaction may be due to the high perceived level of satisfaction resulting from the residents’ perception of a good ecological environment in the national park. Satisfaction has a positive effect on residents’ environmentally responsible behaviors, which is consistent with the findings of Tu [60] et al. Enhancing residents’ satisfaction is necessary to promote the implementation of residents’ environmentally responsible behaviors. The perception of residents’ environmentally responsible behaviors has a significant positive impact on residents’ environmentally responsible behaviors, which is consistent with the findings of Wang [61]. It is necessary to strengthen the publicity and education of ecotourism environmental protection, to improve the level of residents’ ecotourism environmental perception and satisfaction, and to improve environmental responsibility behaviors. So that residents can participate more actively in ecological environmental protection behaviors.

Tourists in China’s Qilian Mountain National Park have a low perception of ecotourism and of residents. Gender differences do not affect the components of the tourist questionnaire. Unlike the residents’ questionnaire, only differences in education and occupation have a significant impact on tourists’ perception of ecotourism environmentally responsible behavior. This is because most of the tourists in the survey are students and corporate employees. Their perception of ecological environmental responsibility may not be affected by age. The correlation verification analysis found a significant positive correlation among several aspects of this study. The perception of ecotourism is an independent positive influencing factor on satisfaction, which indicates that for tourists, strengthening the governance of the ecological environment and creating a comfortable place are the keys to improving tourist satisfaction. The effect of tourists’ perception of environmentally responsible behaviors on satisfaction failed the significance test, which is inconsistent with the findings of Dou [62], probably because the ecological resources in Qilian Mountains National Park have a large deviation from tourists’ expectations, and it is necessary to further explore the cultural connotation of the national park, and to develop and utilize the core resources moderately on the basis of environment protection to enhance tourists’ satisfaction level. Satisfaction has a positive effect on tourists’ environmentally responsible behaviors, probably because tourists’ satisfaction experience with a tourist place can promote the formation of their sense of identity, which in turn can lead to the development of a willingness to protect the environment. The significant positive effect of tourists’ perception of environmental responsibility on tourists’ environmentally responsible behaviors is consistent with the findings of Zhao [63]. The motivation of tourists to consciously participate in environmental protection can be stimulated by enhancing their perceptions of environmental responsibility in national parks.

Through the comparative analysis of residents’ and tourists’ perception of ecotourism environmental responsibility, it is concluded that the perception of residents is more positive and obvious than tourists. It may explain why residents are more aware than tourists of the potential of ecotourism development in China’s Qilian Mountains National Park, and are more optimistic about solving local environmental responsibility problems. Residents and tourists are optimistic about ecotourism. However, residents are dissatisfied with laws and regulations, tourists are neutral, and both residents and tourists have divergence opinions about laws and regulations. The local government has ignored the goal-oriented ecological environmental protection, and the implementation of the ecological protection system was not compulsory and lacked supervision. Both surveyed residents and tourists hold a neutral attitude in their perceptions of residents, but surveyed residents have greater disagreement and tourists have less divergence of opinions about their perceptions of residents, which are inconsistent with the conclusion of Isdell et al. [64] and Hong [57]. Perhaps residents have a better understanding of local issues than tourists do. Some residents may pursue economic benefits while ignoring the ecological and environmental problems caused by economic development.

## 7.Research recommendations

It is important to improve the supervision and management of scenic spots. The level of regulation in Qilian Mountains National Park is closely related to the environmentally responsible behaviors of residents and tourists. The poor maintenance of facilities in some areas of the park has been dangerous. Park managers should provide safe, pleasant, and comfortable ecological sightseeing places for residents and tourists. They should also strengthen the formulation and implementation of scenic spot management norms, encourage residents and visitors to think about their own actions and behaviors through a variety of avenues, strengthen the residents’ and tourists’ perception of environmentally responsible behaviors, change ecologically destructive behaviors, and making ecotourism sustainable.

It is advisable for the government to change the ecological environment concepts and to and promote the balance between ecological environment protection and economic development. The local government sets the tone for ecological environment governance. The results of this study found that the local government paid more attention to economic development than to environmental protection, and some residents were anxious about the balance between revenue and ecological protection. It is necessary for local management departments to enact policies, so that residents can enjoy the benefits of ecotourism while protecting the environment. The government can increase funding, develop agriculture, and help low-income residents participate in the development and construction of ecotourism. The residents of China’s Qilian Mountains National Park should participate in the development of ecotourism and promote environmentally responsible behavior. In addition, the government should establish a robust scientific assessment and evaluation system, enriching innovative supervision, and improving constraints, so that residents and tourists can supervise ecological environmental protection.

It is vital to strengthen publicity and guidance on environmentally responsible behaviors for residents and tourists. The key to promoting the development of ecotourism is to expand the positive impact of ecotourism, and to enhance the perception of residents and tourists. Questionnaire surveys and research results showed that tourists were aware of environmental responsibility, but this awareness may not be reflected in environmentally responsible behaviors, such as the lack of tourists’ participation in ecological environmental protection. In the future, while raising people’s awareness of environmental responsibility, the government should focus on people’s environmentally responsible behaviors, improve the depth and breadth of publicity on ecological and environmental issues, bring publicity closer to the life of residents and tourists, and strengthen their attention to ecological and environmental protection. Establishing a dual tourism environment concept of ecotourism resources and environmental protection in the whole society.

## 8.Research limitations and prospects

Although this study has obtained some important conclusions and enlightenments, there are still some limitations. There are many factors affecting the environmentally responsible behaviors of residents and tourists. The main factors explored in this study cannot fully explain the information, there is a certain incompleteness, and long-term follow-up research should be carried out in the future. Expanding the research perspective and analyzing the characteristics of the impact of ecotourism on the environment at different stages of development, exploring the influence mechanism of various factors on environmentally responsible behaviors, and forming a comprehensive theoretical framework. This research adopts the survey research method of data collection. The data collection tool is the questionnaire filled out by the respondents. Whether respondents based their responses on reality can lead to deviations in study results. Future studies can combine mathematical statistics and quantitative analysis to arrive at more valuable conclusions.

## Supporting information

S1 File (XLSX)

S2 File (XLSX)
